# Real-world pharmacological treatment of patients with hyperemesis gravidarum in 9 cities of China from 2019 to 2024: a cross-sectional analysis

**DOI:** 10.3389/fphar.2026.1828618

**Published:** 2026-05-28

**Authors:** Jiaqian Pan, Jinyuan Wang, Xianli Wang, Tao Zeng

**Affiliations:** 1 Pharmacy Department, Shanghai Key Lab of Reproduction and Development, Shanghai Key Lab of Female Reproductive Endocrine Related Diseases, Obstetrics & Gynecology Hospital of Fudan University, Shanghai, China; 2 Pharmacy Department, Obstetrics and Gynecology Hospital of Fudan University, Yangtze River Delta Integration Demonstration Zone (QingPu), Shanghai, China

**Keywords:** comorbidities, defined daily cost, defined daily doses, hyperemesis gravidarum, prescription analysis

## Abstract

**Objective:**

Analyze the trends and utilization patterns of medicine for hyperemesis gravidarum (HG) in nine Chinese cities over the past 6 years to provide a reference for clinical practice.

**Method:**

The prescription data of drugs for patients diagnosed with HG from 2019–2024 were obtained from the Hospital Prescription Analysis Cooperation Project database of the Hospital Pharmacy Professional Committee of Chinese Pharmaceutical Association. A cross-sectional analysis was then performed of demographic characteristics, medication types, defined daily doses (DDDs), defined daily costs (DDC), combination therapy and comorbidities for HG.

**Results:**

A total of 9097 patients diagnosed with HG, involving 12,505 prescriptions, with a mean age of 29.97 ± 4.35 years. The number of patients in new first-tier cities (Chengdu, Hangzhou, Shenyang, Zhengzhou) exhibited a significant increase. The proportion of advanced maternal age and ≥16 weeks patients has significantly increased. Vitamin B6, metoclopramide, omeprazole, hydrated magnesium hydroxide, and aluminum ranked highest in terms of DDDs. 87% of patients required hospitalization, where combination therapy was commonly observed, predominantly antiemetics combined with antacids/acid reducers. Total prescription costs and *per capita* costs decreased by 42.71% and 55.63% respectively. The gradual decline in the DDC of PPIs and 5-HT3 antagonists may be associated with the implementation of national centralized drug procurement in China. The most common comorbidities for HG in this study included multiple pregnancies, thyroid disease and chronic disease.

**Conclusion:**

From 2019 to 2024, the number of patients with HG increased, while medication costs decreased. This trend is temporally consistent with the progressive implementation of China’s centralized drug-procurement program, which may have contributed to alleviating the economic burden on patients. The average age of patients showed no significant change, while the proportion of advanced maternal age cases exhibited an increasing trend, which may be associated with postponed childbearing intentions. Vitamin B6, omeprazole, and metoclopramide were the most frequently used medications, which consistent with the medication structure recommended by national guidelines. Notably, the usage of ondansetron and metoclopramide has risen significantly, while the frequency of antihistamine use remains lower than that of international standards. Furthermore, multiple pregnancies thyroid dysfunction and chronic disease were the most common comorbidities for HG prescription.

## 1 Introduction

Nausea and Vomiting of Pregnancy (NVP) is typically identified as nausea and/or vomiting during pregnancy within onset before 16 weeks of gestation and in the absence of other identifiable causes. NVP is a common early pregnancy symptom, affecting the quality of life of up to 90% of pregnant women and posing potential threats to maternal and fetal safety (Xu et al., 2024). Hyperemesis gravidarum (HG) represents a severe form of NVP, characterized by complications such as dehydration, metabolic disturbances (weight loss, electrolyte imbalance, or malnutrition), and related complications. HG affects 0.3%–3.6% of pregnant women, severely impairing quality of life and normal dietary intake. In the most extreme situations, the condition may even lead to pregnancy termination (Einarson et al., 2013). If not treated promptly, HG may result in serious complications, including Wernicke encephalopathy (vitamin B1 deficiency-related brain injury), malnutrition, hepatic dysfunction, venous thromboembolism, and even esophageal rupture (Tian et al., 2017; Fejzo et al., 2015; Nozomi Ouchi, 2025).

Current domestic and international guidelines for HG treatment focus primarily on supportive therapies, such as intravenous fluid replacement to correct dehydration and electrolyte imbalances, alongside the use of antiemetic medications to control symptoms (Gadsby et al., 2024). However, there is no universally effective specific therapy, and clinical scenarios such as concomitant gastroesophageal reflux disease (GERD) are common yet insufficiently addressed by guideline recommendations (Pan et al., 2023). Moreover, although the safety of several HG-related medications has been increasingly supported by recent evidence, HG often occurs during early pregnancy when teratogenic risk concerns are heightened, leading to persistent uncertainty among clinicians and patients regarding drug selection. Accordingly, we analyzed a large national prescription dataset to characterize pharmacotherapy patterns in Chinese patients treated for HG. In addition to describing medication utilization, we evaluated economic indicators to estimate drug costs and assessed commonly documented comorbidities to support earlier identification and management of HG populations.

## 2 Data and methods

### 2.1 Data source

Data were obtained from the Hospital Prescription Analysis Cooperation Project of the Hospital Pharmacy Professional Committee of Chinese Pharmaceutical Association. This project collects prescription/medical order data on fixed dates each quarter from nearly 120 hospitals across nine cities (Beijing, Chengdu, Guangzhou, Harbin, Hangzhou, Shanghai, Shenyang, Tianjin, and Zhengzhou). The participating hospitals, distributed across diverse geographical regions, are categorized primary, secondary, and tertiary institutions in accordance with national hospital grading standards. The dataset includes the following information: date, city, route of administration, dose, unit price, dosing frequency, single dose, total cost, patient age, and clinical diagnosis.

### 2.2 Research methods

Outpatient and inpatient prescriptions/medication orders data with clinical diagnosis indicating HG were extracted between 2019 and 2024. The diagnosis of HG used standardized ICD codes. Clinical diagnoses were rigorously established by integrating patient symptoms, physical signs, and laboratory findings in accordance with clinical guidelines (Alshaikh et al., 2026). Data with diagnoses unrelated to HG, such as ectopic pregnancy, infertility, miscarriage were excluded. According to HG treatment guidelines, the primary medications for HG are antiemetics and antacid/acid reducer, yet the safety of using these drugs during pregnancy remains highly controversial (Obstetrics Subgroup, 2015). Therefore, this study specifically focused on antiemetics and antacid/acid reducer, non-relevant treatments were excluded. For instance, intravenous fluid therapy is considered a relatively safe supportive care, while low-molecular-weight heparin is primarily used for prophylaxis and treatment based on specific conditions or VTE risk scores. As such, these treatments are not suitable for direct comparison with the usage patterns of antiemetic/antacid medications in this database.

For further analysis, patients were stratified by age group and region. We screened for the primary therapeutic drugs and conducted detailed analyses based on drug selection, route of administration, dosage, and rationality. According to pharmacological classification, the total prescription cost of drugs was calculated over the 6 years and ranked them by their proportion of the total drug cost of each year.

### 2.3 Comorbidities analysis

Based on guidelines and literature (Pont et al., 2024; Mohammed et al., 2024; Ioannidou et al., 2019), pregnant women with gastrointestinal diseases (*Helicobacter pylori* infection, gastritis), thyroid diseases (thyroid dysfunction, hyperthyroidism), psychiatric disorders (mental disorders, depression), chronic diseases (hypertension, diabetes), and multiple pregnancies are more likely to develop HG. To understand the prevalence of these comorbidities among pregnant women with HG. This study included prescriptions containing these co-documented comorbidities in the “clinical diagnosis” field. Because physicians’ clinical diagnoses may be incomplete, we rigorously analyzed co-documented comorbidities associated with specific disease names and calculated their prevalence.

### 2.4 Evaluation criteria and methods

This study used the Defined Daily Dose (DDD) methodology recommended by the World Health Organization (WHO) (2021) (ATC/DDD index 2022). DDD values were based on the Clinical Medication Guidelines of the Pharmacopoeia of the People’s Republic of China (2020 edition), WHO ATC/DDD data, and drug package inserts. Prescription frequency and costs were calculated for each drug. Furthermore, DDDs and defined daily costs (DDC) were determined. DDDs were computed as the annual total prescribed dose divided by the corresponding DDD. Higher DDDs indicate higher utilization. DDC was computed as the annual total prescription expenditure divided by the DDD for each drug, representing the average daily cost. Larger DDC values indicate greater economic burden. However, drug utilization data presented in DDDs give a rough estimate of consumption and not an exact picture of actual use. Our results should thus be interpreted primarily as trend and relative utilization measures, rather than exact pregnancy-adjusted exposure.

### 2.5 Statistical analysis

Statistical analysis was performed using Excel 2013 and SPSS software, with drugs categorized according to their primary pharmacological actions. We analyzed all included prescriptions and ranked each drug by prescription frequency, cost, DDDs, and DDC. The Mann-Kendall and Cochran-Armitage trend test were applied to assess the statistical significance of trends in quantity and proportions. P < 0.05 was considered statistically significant. To minimize bias, all data were independently evaluated by two pharmacists. Discrepancies were resolved through discussion with a third pharmacist.

## 3 Results

### 3.1 Patient demographics

Based on the inclusion and exclusion criteria, 9,097 patients were included in the study, comprising 1,303 outpatients and 7,794 inpatients. The top three cities for HG patient volume were Zhengzhou (29.33%), Hangzhou (16.58%), and Guangzhou (15.73%), while Harbin and Tianjin reported fewer patients. The average patient age was 29 ± 4.35 years, with approximately 75.84% of patients aged between 25 and 34 years. As the international consensus defines HG as severe nausea or vomiting occurring in early pregnancy (before 16 weeks) (Rhodes et al., 1984). We categorized patients into ≤16 weeks and >16 weeks groups. The proportion of HG patients at 5–16 weeks showed a decreasing trend, while the proportion at 16–36 weeks showed an increasing trend (Table 1).

**TABLE 1:** Demographic analysis.

​	2019	2020	2021	2022	2023	2024	Total	*P*1	*P*2
Region,n (%)									
Beijing	113 (8.32%)	72 (5.33%)	105 (7.17%)	148 (9.69%)	135 (8.21%)	106 (6.05%)	679 (7.46%)	0.462	0.931
Chengdu	95 (7.00%)	97 (7.18%)	100 (6.83%)	126 (8.25%)	170 (10.33%)	169 (9.65%)	757 (8.32%)	0.028↑	<0.001↑
Guangzhou	172 (12.67%)	167 (12.36%)	193 (13.18%)	282 (18.47%)	277 (16.84%)	340 (19.41%)	1,431 (15.73%)	0.086	<0.001↑
Harbin	76 (5.60%)	38 (2.81%)	50 (3.42%)	39 (2.55%)	51 (3.10%)	78 (4.45%)	332 (3.65%)	0.086	<0.001↓
Hangzhou	227 (16.72%)	229 (16.95%)	266 (18.17%)	249 (16.31%)	253 (15.38%)	284 (16.21%)	1,508 (16.58%)	0.028↑	<0.001↓
Shanghai	175 (12.89%)	126 (9.33%)	130 (8.88%)	106 (6.94%)	97 (5.90%)	104 (5.94%)	738 (8.11%)	0.086	<0.001↓
Shenyang	26 (1.91%)	84 (6.22%)	91 (6.22%)	74 (4.85%)	105 (6.38%)	112 (6.39%)	492 (5.41%)	0.028↑	<0.001↑
Tianjin	92 (6.77%)	95 (7.03%)	67 (4.58%)	80 (5.24%)	81 (4.92%)	77 (4.39%)	492 (5.41%)	0.221	<0.001↓
Zhengzhou	382 (28.13%)	443 (32.79%)	462 (31.56%)	423 (27.70%)	476 (28.94%)	482 (27.51%)	2,668 (29.33%)	0.028↑	<0.001↓
Total	1,358 (14.93%)	1,351 (14.85%)	1,464 (16.09%)	1,527 (16.79%)	1,645 (18.08%)	1752 (19.26%)	9097 (100%)	0.027↑	<0.001↑
Drug costs	20839.86 (18.46%)	32116.29 (28.45%)	19783.75 (17.52%)	15106.06 (13.38%)	13118.96 (11.62%)	11939.6 (10.57%)	112904.52 (100%)	0.027↓	<0.001↓
Cost per patient	15.35	23.77	13.51	9.89	7.98	6.81	12.41	0.028↓	​
Age (years)									
18–24	178 (13.11%)	148 (10.96%)	138 (9.43%)	123 (8.06%)	142 (8.63%)	133 (7.59%)	862 (9.48%)	0.086	<0.001↓
25–34	1,010 (74.37%)	1,034 (76.54%)	1,142 (78.01%)	1,135 (74.33%)	1,223 (74.35%)	1,355 (77.34%)	6,899 (75.84%)	0.028↑	0.192
≥35	170 (12.52%)	169 (12.51%)	184 (12.57%)	269 (17.62%)	280 (17.02%)	264 (15.07%)	1,336 (14.69%)	0.086	<0.001↑
Average	29.92 ± 4.35	30.02 ± 4.31	30.14 ± 4.32	30.27 ± 4.36	30.19 ± 4.34	30.16 ± 4.35	29.97 ± 4.35	​	​
Gestational age (week)									
5–15	202 (91%)	197 (87%)	180 (85%)	294 (87%)	364 (89%)	388 (83%)	1,625 (87.41%)	0.086	<0.001↓
16–36	20 (9%)	13 (13%)	33 (15%)	43 (13%)	44 (11%)	81 (17%)	234 (12.59%)	0.027↑	<0.001↑
Total	222 (100%)	210 (100%)	213 (100%)	337 (100%)	408 (100%)	469 (100%)	1859 (100%)	​	​

*P1*, P value for the trend in the number of patients, assessed by the Mann-Kendall trend test; *P2*, P value for the trend in the proportion of patients, assessed by the Cochran-Armitage trend test. The number of patients for each year was the denominator of the data in that column.

### 3.2 DDDs of various drug for HG

Over the 6 years, the DDDs and usage proportions of antacids, dopamine receptor antagonists, and proton pump inhibitors (PPIs) increased significantly, while vitamin DDDs remained relatively stable, but their proportion decreased. The top five drugs by DDDs were Vitamin B6, omeprazole, metoclopramide, hydrotalcite, and esomeprazole. Notably, the DDDs for ondansetron, esomeprazole, pantoprazole, diphenhydramine, and aluminum phosphate increased steadily over time (Table 2).

**TABLE 2:** The DDDs of Anti-hyperemesis gravidarum drugs from 2019 to 2024.

Drug classification	2019	2020	2021	2022	2023	2024	Total	*P1*	*P2*
**Vitamin B6**	**1740.1 (76.62%)**	**1,271.3 (63.67%)**	**1753.1 (66.76%)**	**2028.5 (64.91%)**	**2,148.8 (62.56%)**	**2,117.7 (61.34%)**	**11059.4 (65.42%)**	**0.086**	**<0.001↓**
**Antihistamines**	**12.5 (0.55%)**	**6.5 (0.32%)**	**49.2 (1.87%)**	**28.9 (0.92%)**	**61.1 (1.78%)**	**58.5 (1.69%)**	**216.6 (1.28%)**	**0.221**	**<0.001↑**
Promethazine	9.4 (0.41%)	3.3 (0.16%)	47.1 (1.79%)	9.8 (0.31%)	40.7 (1.18%)	27.6 (0.80%)	137.8 (0.82%)	0.221	<0.001↑
Diphenhydramine	1.6 (0.07%)	3.2 (0.16%)	1.1 (0.04%)	18.6 (0.59%)	20.5 (0.60%)	30.9 (0.89%)	75.8 (0.45%)	0.028**↑**	<0.001↑
Chlorpromazine	1.5 (0.07%)	0.0 (0.00%)	1.0 (0.04%)	0.5 (0.02%)	0.0 (0.00%)	0.0 (0.00)	3.0 (0.02%)	0.043	0.003↓
**Antacid**	**54.3 (1.11%)**	**63.3 (3.36%)**	**83.4 (4.05%)**	**116.5 (5.11%)**	**89.3 (3.92%)**	**104.4 (4.21%)**	**509.4 (4.07%)**	**0.028↑**	**<0.001↑**
Aluminium phosphate	7.9 (0.35%)	23.8 (1.19%)	11.9 (0.45%)	61.5 (1.97%)	63.5 (1.85%)	123.0 (3.56%)	291.7 (1.73%)	0.028**↑**	<0.001↑
Sucralfate	24.0 (1.06%)	61.5 (3.08%)	94.5 (3.60%)	31.1 (1.00%)	105.0 (3.06%)	86.5 (2.51%)	402.6 (2.38%)	0.221	<0.001↑
Almagate	6.4 (0.28%)	5.6 (0.28%)	6.8 (0.26%)	16.1 (0.52%)	11.3 (0.33%)	22.5 (0.65%)	68.6 (0.41%)	0.086	<0.001↑
Hydrotalcite	55.0 (2.42%)	31.6 (1.58%)	74.8 (2.84%)	146.5 (4.69%)	155.0 (4.51%)	94.8 (2.74%)	557.6 (3.30%)	0.086	<0.001↑
**Dopamine receptor antagonists**	**150.8 (6.55%)**	**187.9 (9.97%)**	**256.0 (12.50%)**	**290.1 (13.78%)**	**303.0 (13.33%)**	**246.0 (9.96%)**	**1,432.0 (11.45%)**	**0.028↑**	**<0.001↑**
Metoclopramide	62.5 (2.75%)	77.0 (3.86%)	114.3 (4.35%)	181.2 (5.80%)	167.0 (4.86%)	163.0 (4.72%)	765.0 (4.52%)	0.086	<0.001↑
Domperidone	0.0 (0.00%)	0.0 (0.00%)	0.3 (0.01%)	17.7 (0.57%)	0.0 (0.00%)	6.7 (0.19%)	24.7 (0.15%)	0.130	<0.001↑
**5-HT3 receptor antagonist**	**13.0 (0.75%)**	**23.0 (1.23%)**	**18.0 (0.88%)**	**21.0 (1.00%)**	**31.0 (1.36%)**	**31.0 (1.25%)**	**137.0 (1.10%)**	**0.084**	<0.001↑
Ondansetron	14.0 (0.62%)	20.3 (1.01%)	23.8 (0.90%)	32.5 (1.04%)	57.0 (1.66%)	41.5 (1.20%)	189.0 (1.12%)	0.028**↑**	<0.001↑
Palonosetron	0.0 (0.00%)	3.0 (0.15%)	0.0 (0.00%)	0.0 (0.00%)	0.6 (0.02%)	0.0 (0.00%)	3.6 (0.02%)	0.580	0.006↓
Tropisetron	6.0 (0.26%)	2.0 (0.10%)	0.0 (0.00%)	0.0 (0.00%)	7.0 (0.20%)	1.2 (0.03%)	16.2 (0.10%)	0.086	0.001↓
**Proton pump inhibitor**	**340.2 (14.98%)**	**494.0 (24.74%)**	**497.3 (18.92%)**	**580.5 (18.58%)**	**656.3 (19.11%)**	**734.7 (21.22%)**	**3,303.0 (19.54%)**	**0.028↑**	**<0.001↑**
Ilaprazole	0.0 (0.00%)	0.0 (0.00%)	7.0 (0.27%)	2.0 (0.06%)	0.0 (0.00%)	0.0 (0.00%)	9.0 (0.05%)	0.580	0.850
Omeprazole	243.5 (10.72%)	421.0 (21.09%)	421.0 (16.02%)	511.5 (16.37%)	495.0 (14.41%)	436.0 (12.63%)	2,528.0 (14.95%)	0.130	0.001↓
Esomeprazole	28.7 (1.26%)	20.0 (1.00%)	31.3 (1.19%)	48.0 (1.54%)	113.3 (3.30%)	220.7 (6.39%)	462.0 (2.73%)	0.028**↑**	<0.001↑
Lansoprazole	57.0 (2.51%)	38.0 (1.90%)	13.0 (0.49%)	12.0 (0.38%)	17.0 (0.49%)	35.0 (1.01%)	172.0 (1.02%)	0.221	<0.001↓
Rabeprazole	6.0 (0.26%)	12.0 (0.60%)	19.0 (0.72%)	0.0 (0.00%)	4.0 (0.12%)	0.0 (0.00%)	41.0 (0.24%)	0.130	<0.001↓
Pantoprazole	5.0 (0.22%)	3.0 (0.15%)	6.0 (0.23%)	7.0 (0.22%)	27.0 (0.79%)	43.0 (1.25%)	91.0 (0.54%)	0.028**↑**	<0.001↑
**H2 receptor antagonist**	**2.3 (0.10%)**	**0.0 (0.00%)**	**0.0 (0.00%)**	**0.5 (0.02%)**	**0.3 (0.01%)**	**2.3 (0.07%)**	**5.5 (0.03%)**	**0.130**	**0.513**
Ranitidine	2.3 (0.10%)	0.0 (0.00%)	0.0 (0.00%)	0.0 (0.00%)	0.3 (0.01%)	2.3 (0.07%)	5.0 (0.03%)	0.096	0.408
Roxatidine	0.0 (0.00%)	0.0 (0.00%)	0.0 (0.00%)	0.5 (0.02%)	0.0 (0.00%)	0.0 (0.00%)	0.5 (0.00%)	0.724	0.679
**Glucocorticoid**	**0.03 (0.00%)**	**0.0 (0.00%)**	**0.0 (0.00%)**	**0.0 (0.05%)**	**1.7 (0.05%)**	**0.0 (0.00%)**	**1.7 (0.01%)**	**0.149**	**0.050**
Hydrocortisone	0.03 (0.00%)	0.0 (0.00%)	0.0 (0.00%)	0.0 (0.00%)	1.7 (0.05%)	0.0 (0.00%)	1.7 (0.01%)	0.289	0.050
Total	2,270.9 (13.43%)	1996.6 (11.81%)	2,625.9 (15.53%)	3,124.97 (18.49%)	3,434.6 (20.32%)	3,452.3 (20.42%)	16905.3 (100%)	0.009↑	<0.001↑

DDDs: defined daily doses = the total annual prescribed dose of drugs/ the DDD, of drugs. *P1*, P value for the trend in the DDDs, of Anti-hyperemesis gravidarum drugs, assessed by the Mann-Kendall trend test; *P2*, P value for the trend in the proportion of DDDs, assessed by the Cochran-Armitage trend test. The total DDDs, for each year was the denominator of the data in that column. Bold text represents the classification of drugs.

### 3.3 Economic analysis of various drug for HG

Total HG prescription costs decreased by 42.7%, from RMB 20,839.86 in 2019 to RMB 11,939.60 in 2024 (Table 3). Per-patient costs decreased from RMB 15.35 to RMB 6.81 (Table 1). The top five drug categories by total cost were PPIs, vitamins, 5-HT_3_ antagonists, dopamine receptor antagonists, and antacids. The top five specific drugs were omeprazole, vitamin B6, lansoprazole, ondansetron, and metoclopramide. Furthermore, the costs and proportions for diphenhydramine, ondansetron, esomeprazole, and pantoprazole increased year by year, showing statistical significance (Table 3).

**TABLE 3:** The cost of Anti-hyperemesis gravidarum drugs from 2019 to 2024.

Drug classification	2019	2020	2021	2022	2023	2024	Amount (RMB)	*P1*	*P2*
**Vitamin B6**	**4,202.9 (20.17%)**	**3,649.3 (11.36%)**	**3,826.5 (19.34%)**	**4,287.2 (28.38%)**	**4,638.1 (35.35%)**	**2,637.0 (22.09%)**	**23240.9 (20.58%)**	**0.221**	**<0.001↑**
**Antihistamines**	**87.1 (0.42%)**	**93.7 (0.29%)**	**114.5 (0.58%)**	**453.2 (3.00%)**	**585.9 (4.47%)**	**1,199.5 (10.05%)**	**2,533.9 (2.24%)**	**0.028↑**	**<0.001↑**
Promethazine	48.4 (0.23%)	20.8 (0.06%)	86.1 (0.43%)	44.5 (0.29%)	61.6 (0.47%)	134.9 (1.13%)	396.2 (0.35%)	0.221	<0.001↑
Diphenhydramine	34.8 (0.17%)	72.9 (0.23%)	25.8 (0.13%)	407.3 (2.70%)	524.4 (4.00%)	1,064.7 (8.92%)	2,129.8 (1.89%)	0.028↑	<0.001↑
Chlorpromazine	3.9 (0.02%)	0.0 (0.00%)	2.6 (0.01%)	1.4 (0.01%)	0.0 (0.00%)	0.0 (0.00%)	7.9 (0.01%)	0.043↓	0.143
**Antacid**	**497.4 (2.39%)**	**540.3 (1.68%)**	**641.4 (3.24%)**	**707.6 (4.68%)**	**848.8 (6.47%)**	**835.3 (7.00%)**	**4,070.8 (3.61%)**	**0.028↑**	**<0.001↑**
Aluminium phosphate	18.6 (0.09%)	55.9 (0.17%)	24.5 (0.12%)	117.8 (0.78%)	121.6 (0.93%)	257.5 (2.16%)	596.0 (0.53%)	0.028↑	<0.001↑
Sucralfate	72.2 (0.35%)	196.0 (0.61%)	296.5 (1.50%)	87.9 (0.58%)	318.1 (2.42%)	246.0 (2.06%)	1,216.7 (1.08%)	0.221	<0.001↑
Almagate	33.0 (0.16%)	58.2 (0.18%)	34.9 (0.18%)	84.1 (0.56%)	58.2 (0.44%)	117.2 (0.98%)	385.7 (0.34%)	0.086	<0.001↑
Hydrotalcite	373.6 (1.79%)	230.1 (0.72%)	285.5 (1.44%)	417.8 (2.77%)	350.9 (2.67%)	214.52 (1.80%)	1872.4 (1.66%)	0.462	<0.001↑
**Dopamine receptor antagonists**	**429.7 (2.06%)**	**399.9 (1.25%)**	**552.3 (2.79%)**	**904.9 (5.99%)**	**1,011.8 (7.71%)**	**896.5 (7.51%)**	**4,195.2 (3.72%)**	**0.086**	**<0.001↑**
Metoclopramide	429.7 (2.06%)	399.9 (1.25%)	551.8 (2.79%)	894.3 (5.92%)	1,011.8 (7.71%)	893.3 (7.48%)	4,180.9 (3.70%)	0.086	<0.001↑
Domperidone	0.0 (0.00%)	0.0 (0.00%)	0.5 (0.00%)	10.6 (0.07%)	0.0 (0.00%)	3.2 (0.03%)	14.3 (0.01%)	0.13	0.042↑
**5-HT3 receptor antagonist**	**885.4 (4.25%)**	**1,457.1 (4.54%)**	**1,159.7 (5.86%)**	**1,343.4 (8.89%)**	**1939.2 (14.78%)**	**1,324.2 (11.09%)**	**81089.0 (7.18%)**	**0.221**	**<0.001↑**
Ondansetron	495.4 (2.38%)	890.8 (2.77%)	1,159.7 (5.86%)	1,343.7 (8.89%)	1923.2 (14.66%)	1,323.0 (11.08%)	7,135.5 (6.32%)	0.028↑	<0.001↑
Palonosetron	0.0 (0.00%)	422.8 (1.32%)	0.0 (0.00%)	0.0 (0.00%)	4.8 (0.04%)	0.0 (0.00%)	427.6 (0.38%)	0.579	<0.001↓
Tropisetron	390.0 (1.87%)	143.5 (0.45%)	0.0 (0.00%)	0.0 (0.00%)	11.3 (0.09%)	1.2 (0.01%)	545.9 (0.48%)	0.221	<0.001↓
**Proton pump inhibitor**	**14728.6 (70.67%)**	**25976.1 (80.88%)**	**13489.4 (68.18%)**	**7,230.9 (47.87%)**	**3,898.5 (29.72%)**	**4,713.9 (39.48%)**	**70037.3 (62.03%)**	**0.086**	**<0.001↓**
Ilaprazole	0.0 (0.00%)	0.0 (0.00%)	1,092.0 (5.52%)	142.0 (0.94%)	0.0 (0.00%)	0.0 (0.00%)	1,234.0 (1.09%)	0.579	<0.001↓
Omeprazole	8,609.7 (41.31%)	19564.2 (60.92%)	8,185.3 (41.37%)	6,696.9 (44.33%)	3,129.0 (23.85%)	3,771.4 (31.59%)	49956.4 (44.25%)	0.086	<0.001↓
Esomeprazole	1,407.8 (6.76%)	1744.6 (5.43%)	1,669.4 (8.44%)	348.9 (2.31%)	566.3 (4.32%)	738.7 (6.19%)	6,475.7 (5.74%)	0.462	<0.001↓
Lansoprazole	3,897.3 (18.70%)	2,813.8 (8.76%)	686.4 (3.47%)	21.9 (0.14%)	40.3 (0.31%)	151.5 (1.27%)	7,611.2 (6.74%)	0.086	<0.001↓
Rabeprazole	690.3 (3.31%)	1,277.9 (3.98%)	1,588.1 (8.03%)	0.0 (0.00%)	33.5 (0.26%)	0.0 (0.00%)	3,589.8 (3.18%)	0.312	<0.001↓
Pantoprazole	123.4 (0.59%)	575.6 (1.79%)	268.2 (1.36%)	21.1 (0.14%)	129.4 (0.99%)	52.4 (0.44%)	1,170.2 (1.04%)	0.221	<0.001↓
**H2 receptor antagonist**	**8.4 (0.04%)**	**0.0 (0.00%)**	**0.0 (0.00%)**	**171.1 (1.13%)**	**47.6 (0.36%)**	**333.2 (2.79%)**	**560.4 (0.50%)**	**0.13**	**<0.001↑**
Ranitidine	8.4 (0.04%)	0.0 (0.00%)	0.0 (0.00%)	0.0 (0.00%)	47.6 (0.36%)	333.2 (2.79%)	389.2 (0.34%)	0.096	<0.001↑
Roxatidine	0.0 (0.00%)	0.0 (0.00%)	0.0 (0.00%)	171.1 (1.13%)	0.0 (0.00%)	0.0 (0.00%)	171.1 (0.15%)	0.724	0.002↑
**Glucocorticoid**	**0.4 (0.00%)**	**0.0 (0.00%)**	**0.0 (0.00%)**	**7.9 (0.05%)**	**149.0 (1.14%)**	**0.0 (0.00%)**	**157.2 (0.14%)**	**0.312**	**<0.001↑**
Hydrocortisone	0.4 (0.00%)	0.0 (0.00%)	0.0 (0.00%)	0.0 (0.00%)	149.0 (1.14%)	0.0 (0.00%)	149.4 (0.13%)	0.289	<0.001↑
Hydrocortisone sodium succinate	0.0 (0.00%)	0.0 (0.00%)	0.0 (0.00%)	7.9 (0.05%)	0.0 (0.00%)	0.0 (0.00%)	7.9 (0.01%)	0.724	0.513
Total	20839.9 (100.00%)	32116.3 (100.00%)	19783.8 (100.00%)	15106.1 (100.00%)	13119.0 (100.00%)	11939.6 (100.00%)	112904.5 (100.00%)	0.028↓	​

*P1*, P value for the trend in the cost of Anti-hyperemesis gravidarum drugs, assessed by the Mann-Kendall trend test; *P2*, P value for the trend in the proportion of cost, assessed by the Cochran-Armitage trend test. The total cost for each year was the denominator of the data in that column. Bold text represents the classification of drugs.

### 3.4 DDC of various drug for HG

From 2019 to 2021, PPIs (rabeprazole, pantoprazole, and ilaprazole) had the highest DDC values respectively; from 2022 onward, H2 receptor antagonists (roxatidine and ranitidine) ranked highest respectively. The DDC of omeprazole decreased markedly starting in 2021. DDC for esomeprazole, lansoprazole, rabeprazole, and pantoprazole decreased significantly beginning in 2022. The DDC of ondansetron did not change significantly over the 6 years, but its ranking rose to third place by 2022 (Table 4).

**TABLE 4:** DDC of Anti-hyperemesis gravidarum drugs from 2019 to 2024.

Rank	2019		2020		2021		2022		2023		2024	
	Generic name	DDC (yuan)	Generic name	DDC (yuan)	Generic name	DDC (yuan)	Generic name	DDC (yuan)	Generic name	DDC (yuan)	Generic name	DDC (yuan)
1	Rabeprazole	115.06	Pantoprazole	191.88	Ilaprazole	156.00	Roxatidine	342.26	Ranitidine	158.67	Ranitidine	144.87
2	Lansoprazole	68.37	Palonosetron	140.95	Rabeprazole	83.59	Ilaprazole	71.00	Hydrocortisone	87.65	Diphenhydramine	34.46
3	Tropisetron	65.00	Rabeprazole	106.49	Esomeprazole	53.33	Ondansetron	41.33	Ondansetron	33.74	Ondansetron	31.88
4	Esomeprazole	49.05	Esomeprazole	87.23	Lansoprazole	52.80	Diphenhydramine	21.90	Diphenhydramine	25.58	Omeprazole	8.65
5	Ondansetron	35.39	Lansoprazole	74.05	Ondansetron	48.73	Omeprazole	13.09	Rabeprazole	8.38	Metoclopramide	5.48
6	Omeprazole	35.36	Tropisetron	71.73	Pantoprazole	44.70	Esomeprazole	7.27	Palonosetron	7.97	Almagate	5.21
7	Pantoprazole	24.69	Omeprazole	46.47	Diphenhydramine	23.49	Almagate	5.23	Omeprazole	6.32	Promethazine	4.89
8	Diphenhydramine	21.73	Ondansetron	43.88	Omeprazole	19.44	Metoclopramide	4.94	Metoclopramide	6.06	Lansoprazole	4.33
9	Hydrocortisone	11.67	Diphenhydramine	22.77	Almagate	5.14	Promethazine	4.54	Almagate	5.15	Esomeprazole	3.35
10	Metoclopramide	6.87	Almagate	10.39	Metoclopramide	4.83	Pantoprazole	3.02	Esomeprazole	5.00	Sucralfate	2.84

DDC: defined daily cost = total annual prescription amount of drugs/DDDs, of drugs.

### 3.5 Medication patterns

**FIGURE 1:**
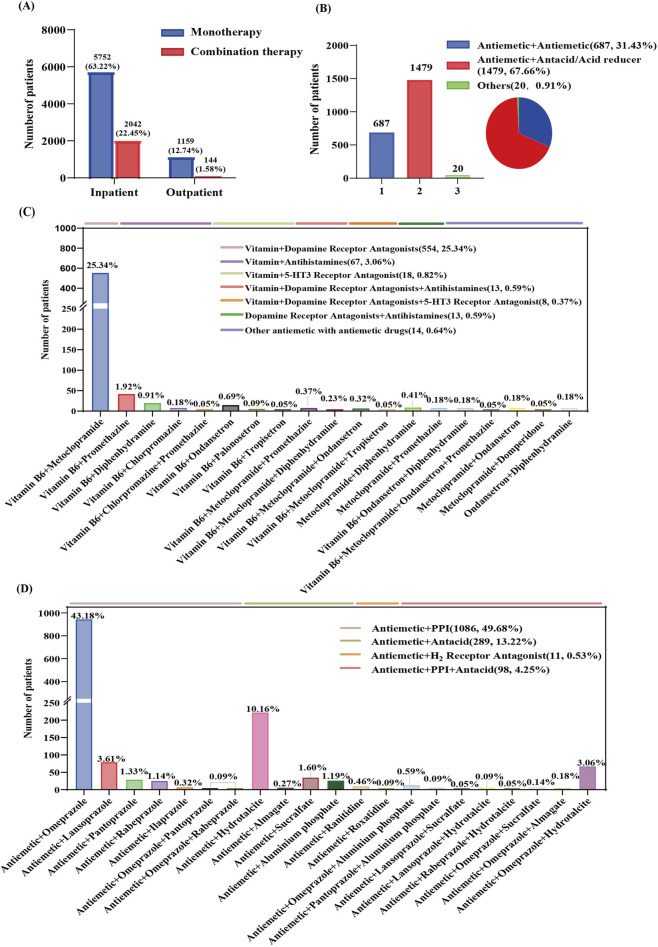
The situation of Anti-hyperemesis gravidarum drugs for HG patients from 2019 to 2024. **(A)** Classification of Anti-hyperemesis gravidarum drugs in Inpatient/Outpatient; **(B)** Classification of combined therapy; **(C)** The combined therapy of antiemetic drugs; **(D)** The combined therapy of antiemetic with antacids/acid reducer.

Monotherapy was administered to 76% of HG patients, while the remaining 24% received combination therapy involving 109 distinct combinations (Figure 1A). Combination therapy was primarily utilized in patients unresponsive to single antiemetics or those with vomiting complicated by GERD. The combined therapies can be mainly classified into two groups: antiemetic combinations and antiemetics + antacids/acid reducers (Figure 1B). Antiemetics included vitamins, antihistamines, dopamine receptor antagonists, and 5-HT_3_ receptor antagonists (Figure 1C). Antacids included aluminum phosphate, sucralfate, almagate, and hydrotalcite; acid reducers mainly included PPIs and H2 receptor antagonists (Figure 1D). The top five combinations were: Antiemetic + PPI, Vitamins + Dopamine receptor antagonist, Antiemetic + Antacid, Antiemetic + PPI + Antacid, and Vitamins + Antihistamine.

### 3.6 Comorbidities related to HG

**FIGURE 2:**
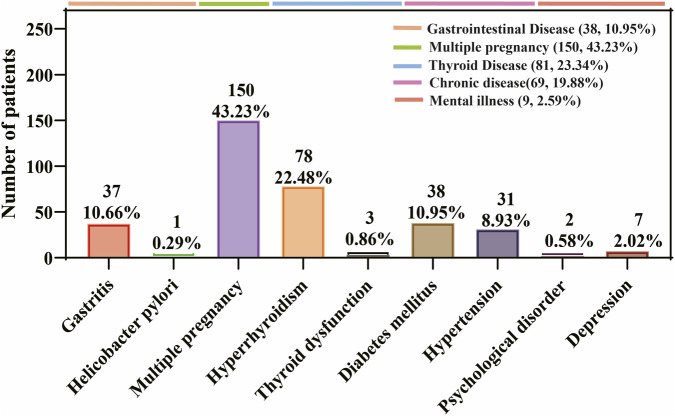
Comorbidities of HG

We found 347 HG patients with comorbidities recorded in the “clinical diagnosis” field, accounting for 2.77% of prescriptions. These included gastrointestinal diseases, multiple pregnancies, thyroid diseases, chronic diseases, and mental illness. Multiple pregnancies, hyperthyroidism and chronic diseases were the most common comorbidities (Figure 2).

## 4 Discussion

### 4.1 Demographic characteristics

Over the past 6 years, both the number and proportion of patients treated for HG have increased significantly (P < 0.001). This suggests a growing awareness of seeking medical attention among women with HG. Additionally, rising living costs and social pressures may increase psychological stress during pregnancy, potentially contributing to a higher occurrence of HG (Kjeldgaard et al., 2017). Regionally, new first-tier cities (Hangzhou and Zhengzhou) exhibited higher HG treatment volumes than first-tier cities (Beijing, Shanghai, and Guangzhou), whereas some second-tier cities showed lower volumes (Harbin, Shenyang, and Tianjin). The volume of patients across Chengdu, Hangzhou, Shenyang, and Zhengzhou pronounced surge. Such regional variations may be related to the distribution of medical resources and diagnostic levels. Major urban centers may have more comprehensive recognition, diagnosis, and documentation of HG, leading to higher recorded case counts.

The age distribution was concentrated in the typical reproductive range, with most patients aged 25–44 years. Although the number of older pregnant women (>34 years) showed no clear trend, their proportion increased significantly. Although recent retrospective analyses suggest younger mothers are at higher risk for HG (Ioannidou et al., 2019), our study had a small base of patients aged 18–24, preventing definitive conclusions regarding age and risk. Future studies are needed to clarify the relationship between maternal age and HG.

### 4.2 Drug categories

Analysis of outpatient and inpatient prescriptions reveals that the first-line agent Vitamin dominates due to its high safety profile, often used as a foundational drug in combination therapies. PPIs and dopamine receptor antagonists followed, mainly concentrated in inpatient settings. Recent systematic reviews and meta-analyses (Gill et al., 2009; Li et al., 2020) indicate no significant association between maternal PPI use and adverse neonatal outcomes, suggesting they are likely safe in pregnancy. Although PPIs are frequently prescribed, they are typically administered as adjunctive therapy with antiemetics rather than as first-line monotherapy for HG. An observational study (Gill et al., 2009) found that, for patients with heartburn/acid reflux, concomitant acid-suppressing therapy (e.g., antacids, H2 receptor antagonists and PPIs) together with antiemetic treatment can significantly improve symptoms and overall health status within 3–4 days. It is often difficult to determine whether gastroesophageal acid reflux is a triggering factor in patients with severe HG. Therefore, clinicians commonly initiate prophylactic acid-suppressing therapy for such patients. For HG patients with GERD unresponsive to antiemetics, the addition of first-generation PPIs (omeprazole, lansoprazole, pantoprazole) is recommended. However, long-term PPI use may increase the risk of acute kidney injury and end-stage renal disease, necessitating caution in patients with kidney disease (Toth-Manikowski and Grams, 2017). Prescriptions for metoclopramide increased significantly. Adverse reactions to metoclopramide in pregnancy are generally mild, mostly associated with continuous subcutaneous injections, including drowsiness, injection site irritation, restlessness, and extrapyramidal symptoms (EPS) (Wu and Ma, 2024). However, a recent systematic review and meta-analysis on metoclopramide use included a study showing a significant association with overall major congenital malformations (aOR: 1.27; 1.03–1.57) and genital malformations (aOR: 2.26; 1.14–4.48) (Sun et al., 2021). Therefore, vigilance for potential genital malformations is warranted. Utilization of the 5-HT3 antagonist ondansetron increased year by year. This trend may be related to accumulating evidence that ondansetron is generally safe and does not substantially increase congenital malformation risk (Huybrechts et al., 2020; Lemon et al., 2020; Huybrechts et al., 2018). A comparative study found that women not taking ondansetron had higher rates of pregnancy termination, while those taking it had higher live birth rates (Dormuth et al., 2021). Furthermore, past studies suggest that ondansetron has superior antiemetic efficacy compared to metoclopramide (Kashifard et al., 2013). Therefore, ondansetron is a widespread clinical adoption as second-line therapy.

### 4.3 Analysis of combination therapy patterns

We analyzed the primary combination patterns for HG over the past 6 years. Antiemetic and PPI combinations accounted for 50% of all combination prescriptions; omeprazole was the most used PPI, an appropriate option for HG complicated by GERD. The combination of vitamins and the dopamine receptor antagonist metoclopramide accounted for 25%. Although specific efficacy/safety studies for this combination are lacking, current guidelines argue metoclopramide safe and effective, recommending its combination with other antiemetics. Combinations of antiemetics and antacids (hydrotalcite, sucralfate, aluminum phosphate, almagate) ranked third. Hydrotalcite is preferred: while traditional carbonate antacids are potent, they may have adverse effects on the fetus/neonate (Mahadevan, 2007). Aluminum/magnesium-containing antacids are safer but carry risks of electrolyte disturbance with long-term use. Hydrotalcite offers advantages in both antacid efficacy and safety, making it suitable for long-term use in HG. Vitamins combined with antihistamines (promethazine, diphenhydramine) were also observed. A controlled trial reported that combining vitamin B6 with promethazine significantly improved HG treatment outcomes (Deng et al., 2025). Although guidelines recommend promethazine as a first-line antiemetic, clinical practice sees wider use of second-line metoclopramide combined with vitamins. A study of 530 admissions for HG from UK (Fiaschi et al., 2019) demonstrated that cyclizine was the most frequently prescribed (almost 73% of admissions). Notably, the usage rate of antihistamines in this study was 1.28%. This aligns with the rationale under national guidelines (Obstetrics Subgroup, 2015), but is lower than the levels recommended by international guidelines. A possible reason for this discrepancy is that while antihistamines (doxylamine) are recommended as a first-line treatment in international guidelines (Gadsby et al., 2024), doxylamine is not available in the Chinese market. Additionally, the combination of ondansetron and vitamin B6 ranked fourth. A study of 1,037 HG admissions (Fiaschi et al., 2019) indicated that combinations of dopamine and 5-HT3 antagonists with other antiemetics were common in readmitted patients, though specific efficacy studies are pending. Clinicians must comprehensively assess severity, safety, and interactions when deciding on combination therapy. Future research should evaluate optimal combination regimens to improve symptom control while minimizing pregnancy-related risks.

### 4.4 Pharmacoeconomic evaluation

The substantial decline in total prescription costs from 2019 to 2024 is plausibly associated with the progressive implementation of China’s centralized drug-procurement Program. Oral omeprazole was included in the China centralized drug-procurement Program in 2020, followed by a marked reduction in its DDC by the following year. In 2021, esomeprazole, pantoprazole, lansoprazole, and injectable omeprazole were included, subsequently, a significant DDC decrease for PPIs in 2022. Esomeprazole, the S-isomer of omeprazole, offers higher bioavailability (77%–90%) and more stable acid suppression. Its usage frequency has increased annually, ranking third in DDDs by 2024, following omeprazole. Expensive 5-HT_3_ antagonists like palonosetron and tropisetron were used less frequently but were relatively costly; Their DDC values decreased substantially from 2021,which may be linked to its inclusion in procurement in 2021. Although injectable ondansetron was included in 2022, the oral dosage form has not yet been included, which may explain the absence of a clear DDC decrease during the study period. In addition, we calculated only medication costs and did not specifically analyze differences between oral and injectable formulations. However, injectables formulations generally require more complex manufacturing processes, and injectables formulations are often accompanied by hospitalization-related expenses and nursing/administration costs. The overall costs of injectables formulations are usually higher in practice.

### 4.5 Analysis of HG comorbidities

Our analysis found multiple pregnancies (43.23%), thyroid disease (23.34%) and chronic disease (19.88%) as the common comorbidities, consistent with previous studies (Pont et al., 2024; Mohammed et al., 2024; Ioannidou et al., 2019). Deshayne B et al. (Fell et al., 2006) identifed pregnant women with diabetes and mental illness are more likely to develop HG though the mechanisms remain unclarified. The results of this study showed that some patients with HG also suffer from hypertension, diabetes, and mental illness. However, due to the limited sample size, no correlation analysis between HG and these comorbidities was performed. Nevertheless, Future clinical management of HG should prioritize early identification and education for pregnant women with these comorbidities to improve maternal and infant health outcomes.

## 5 Conclusion

Based on prescriptions/orders for HG patients from 2019 to 2024, this study found a significant upward trend in the number of patients treated, reflecting increased medical-seeking behavior among women of childbearing age. Regarding pharmacotherapy, vitamins, PPIs, and dopamine receptor antagonists remain the core treatments, with overall rational usage compared with national guidelines. The utilization of metoclopramide and ondansetron is increasing annually, which may positively influence pregnancy outcomes and delivery rates. However, compared to international guidelines, the application of antihistamines in China is low. Combination regimens should be individualized based on disease severity and medication availability rather than relying on a fixed approach. The National-Organized Centralized Procurement of Drugs Policy has effectively reduced HG medication costs. We suggest progressively including high-frequency, high-cost drugs like oral ondansetron and diphenhydramine into the procurement program to further mitigate patient burden. Additionally, identifying comorbidities such as thyroid dysfunction and gastrointestinal diseases provides a critical basis for early intervention in high-risk groups. In summary, our findings align with national consensus, detailing the status and trends of HG medication, and offer guidance for safe, rational clinical practice. However, this study has certain limitations. The data were exclusively sourced from participating hospitals across only nine cities, which may not fully reflect the nationwide usage. Future studies could further expand the representativeness of the sample and leverage real-world data to conduct more comprehensive research on the use of HG.

## Data Availability

The original contributions presented in the study are included in the article/supplementary material, further inquiries can be directed to the corresponding authors.
